# Comorbid psoriasis in systemic lupus erythematosus: a cohort study from a tertiary referral centre and the National Patient Register in Sweden

**DOI:** 10.1136/lupus-2025-001504

**Published:** 2025-06-04

**Authors:** Tomas Walhelm, Ioannis Parodis, Charlotta Enerbäck, Elizabeth Arkema, Christopher Sjöwall

**Affiliations:** 1 Department of Biomedical and Clinical Sciences, Division of Inflammation and Infection/Rheumatology, Linköping University, Linköping, Sweden; 2 Division of Rheumatology, Department of Medicine Solna, Karolinska Institutet, Karolinska University Hospital, and Center for Molecular Medicine (CMM), Stockholm, Sweden; 3 Department of Rheumatology, Faculty of Medicine and Health, Örebro University, Örebro, Sweden; 4 Ingrid Asp Psoriasis Research Center, Department of Biomedical and Clinical Sciences, Linköping University, Linköping, Sweden; 5 Department of Medicine Solna, Clinical Epidemiology Division, Karolinska Institutet, Stockholm, Sweden

**Keywords:** Lupus Erythematosus, Systemic, Epidemiology, Treatment, Autoantibodies

## Abstract

**Objectives:**

To investigate the prevalence of psoriasis in SLE using a Swedish regional cohort and a nationwide cohort from the National Patient Register (NPR). Furthermore, we compared clinical features between patients with and without comorbid psoriasis.

**Methods:**

In total, 351 patients diagnosed with SLE based on the 1982 American College of Rheumatology and/or the 2012 Systemic Lupus International Collaborating Clinics criteria from Linköping University Hospital were evaluated. We obtained patient-reported and relevant clinical data extracted in 2024. Individuals with coexisting psoriasis were identified via the International Classification of Diseases code L40 and subsequent confirmation through chart review in the regional cohort. In the NPR, 7490 subjects with SLE living in Sweden in 2022 were identified, as well as therapies obtained from the Prescribed Drug Register.

**Results:**

We identified 12 subjects with SLE and coexisting psoriasis (3.4%) in the regional cohort and 367 patients (4.9%) in the nationwide cohort. Men were proportionally more common in the group with comorbid psoriasis in both cohorts. Patients with psoriasis reported more pain on a visual analogue scale (median 45.5/100 mm, IQR 23.3–58.3) compared with those without coexisting psoriasis (median 27.0/100 mm, IQR 7.0–50.5, p<0.04). We observed no differences in damage accrual or clinical phenotypes between the two groups. Subjects with psoriasis were more frequently prescribed methotrexate in the nationwide cohort.

**Conclusion:**

The prevalence of coexisting psoriasis in patients with SLE in Sweden was estimated to be 3.4–4.9%. Individuals with comorbid psoriasis reported more pain and were more likely to be prescribed methotrexate than those without psoriasis.

**WHAT IS ALREADY KNOWN ON THIS TOPIC**

- Patients with SLE are at an increased risk of developing additional autoimmune disorders. The coexistence of SLE and psoriasis has been described in case reports and smaller cohort studies, but not in nationwide studies.

**WHAT THIS STUDY ADDS**

- The co-occurrence of SLE and psoriasis was reported in 3.4% of patients with confirmed SLE and in 4.9% of patients with SLE in the Swedish National Patient Register. Patients with comorbid psoriasis reported more pain using a visual analogue scale and were more frequently treated with methotrexate, according to Swedish Prescribed Drug Register data. The percentage of male sex was higher among patients with SLE with coexisting psoriasis, and this SLE patient subgroup also showed trends towards higher exposure to tobacco and higher prevalence of anti-U1-ribonucleoprotein antibodies.

**HOW THIS STUDY MIGHT AFFECT RESEARCH, PRACTICE OR POLICY**

- In patients with SLE living in Sweden, psoriasis co-occurs in approximately 4%, emphasising the importance of regular screening of patients with SLE for other coexisting autoimmune diseases. Comorbid psoriasis in patients with SLE may have implications for the choice of treatment.

## Introduction

 SLE is a complex autoimmune disease with variable clinical presentations, including musculoskeletal and mucocutaneous manifestations, haematological abnormalities, and kidney and nervous system involvement.^1^ Psoriasis is a chronic systemic inflammatory disease that affects the skin and nails.^2^ While diagnostic criteria for psoriatic arthritis (PsA) have not been validated, the 2006 classification criteria define PsA for the purpose of enrolling patients in clinical trials and provide diagnostic guidance to clinicians.^3^ Over time, up to 30% of patients with psoriasis develop PsA.^3^

While key cytokines and driving mechanisms are shared between SLE and psoriasis, the two conditions also have some distinct underlying pathophysiological mechanisms.^4 5^ In addition, shared loci conferring genetic susceptibility to the two conditions, including tumour necrosis factor (TNF) receptor-associated factor 3 interacting protein 2,^6 7^ signal transducer and activator of transcription 4 (STAT4)^8 9^ and protein tyrosine phosphatase non-receptor type 22, have been reported.^10 11^ Despite dissimilar aspects of their pathogenesis, psoriasis and SLE may have phenotypical similarities.^12^ The psoriasiform lesions of subacute cutaneous lupus erythematosus, as well as the more prevalent discoid lupus lesions, have overlapping features with psoriasis and can cause diagnostic challenges.^13^

Some pharmacological treatments are highly relevant to patients with both SLE and psoriasis. Methotrexate is frequently used to treat SLE with active joint or skin involvement, and it is also effective in managing psoriasis.^14 15^ However, some drugs used in SLE are known for their capacity to trigger the development of psoriasis and should be avoided. For example, the cornerstone agent for SLE, hydroxychloroquine, has been reported to induce or trigger relapses of psoriasis.^16^ TNF inhibitors (eg, etanercept and adalimumab) represent standard treatments for psoriasis, but are contraindicated in SLE.

The estimated prevalence of psoriasis among the adult population in Sweden is 2.1%,^17^ but its concurrent occurrence with SLE remains uncertain. Coexistence of SLE and psoriasis has been reported in case reports and in a cohort study from Canada.^18^,^20^ That study found psoriasis in approximately 3.5% of an SLE population, which notably was twice as high compared with the general Canadian population.

The objective of the current study was to investigate the coexistence of psoriasis in a well-defined Swedish regional cohort and to, in detail, compare patients with SLE with comorbid psoriasis to patients with SLE without psoriasis regarding clinical variables, serological characteristics, patient-reported outcome measures (PROMs) and treatment strategies. We next enlarged our study population by using the National Patient Register (NPR) to identify individuals bearing International Classification of Diseases Tenth Revision (ICD-10) codes for SLE with and without psoriasis.

## Methods

### Study population

The regional Swedish cohort *Clinical Lupus Register in Northeastern Gothia* (Swedish acronym: KLURING) was used to determine the clinical and immunological characteristics of patients with SLE. All patients in KLURING were adults (≥18 years old) who met the validated 1982 American College of Rheumatology (ACR) and/or the 2012 Systemic Lupus International Collaborating Clinics (SLICC) classification criteria for SLE.^21 22^ KLURING was launched in 2008 and a detailed overview of the cohort was recently published.^23^ PROMs are collected using the 3-level version of EQ-5D,^24^ 100 mm visual analogue scales (VAS) for pain, fatigue and SLE-related overall health experience, and the Health Assessment Questionnaire Disability Index.^25^ Disease activity is assessed using the clinical version of SLE Disease Activity Index 2000 (cSLEDAI-2K) and a 4-point scale for the Physician’s Global Assessment.^26 27^ Organ damage is annually evaluated using the SLICC/ACR Damage Index (SDI).^28^ Smoking status, treatments and body mass index are registered at each visit, along with other relevant clinical and routine laboratory data. PROMs (median) and SDI (mean) data were extracted with values of the three last available measurements; clinical parameters and manifestations were extracted with data from the last available visit.^29^

Detailed immune serology was collected at last follow-up, including ANA (immunofluorescence technique) and antibodies against double-stranded DNA, Smith antigen, U1-ribonucleoprotein (anti-U1-RNP), Sjögren’s syndrome A (anti-SSA/Ro52 and anti-SSA/Ro60), Sjögren’s syndrome B (anti-SSB/La), cardiolipin, β2-glycoprotein-I and cyclic citrullinated peptide.^30 31^ Additionally, data on the lupus anticoagulant test, rheumatoid factor and C3 and C4 levels were collected. Serological characteristics were extracted from the KLURING cohort in 2024, and autoantibody positivity on an ever basis was used in the statistical calculations.

To identify patients with coexisting psoriasis in KLURING, a search was conducted in the electronic medical records for the ICD-10 code: L40. For all patients with an ICD-10 L40 code, a thorough chart review was conducted to determine the reliability of the diagnosis, type of psoriasis and history of treatment for psoriasis. The data extraction from the cohort and the medical charts was performed in June 2024.

### National register data

The NPR contains information on visits in inpatient care (since 1964, nationwide since 1987) and outpatient specialist care (since 2001) in Sweden. Individuals were considered to have SLE if they had at least two visits listing M32 (excluding M32.0), with at least one visit in a clinic that typically treats SLE (rheumatology, internal medicine, dermatology, nephrology, paediatrics). This SLE definition is estimated to have a positive predictive value of 80.1%.^32^ Of these patients, we included those who were adults (aged 18 or older), living in Sweden as of 31 December 2022. Patients with a visit listing a psoriasis diagnosis (ICD-10 L40) were considered to have comorbid psoriasis. In a sensitivity analysis, we defined psoriasis as having at least one visit listing an ICD code for psoriasis or at least one dispensation of a topical vitamin D analogue (Anatomical Therapeutic Chemical (ATC) code D05AX02 or D05AX52).

The Swedish Prescribed Drug Register (PDR) includes information on all dispensations for prescribed drugs in pharmacies in Sweden since July 2005. ATC codes were used to identify medications dispensed in the 2 years before inclusion (January 2021 to December 2022) and since the inception of the PDR (since July 2005). The Total Population Register provided information on birth date (to calculate age), sex and residence (to determine if living in Sweden on 31 December 2022). Data were linked across the registers using each individual’s personal identification number.

### Statistics

Descriptive statistics are presented as medians and the corresponding IQR or means with the SD. Categorical data regarding clinical manifestations and immunological abnormalities between patients with coexisting psoriasis compared with patients with SLE without psoriasis were examined using the χ^2^ test or the Fisher’s exact test as appropriate. For comparisons of continuous data, the Mann-Whitney U test was used when the distributions were not normal, while the Student’s t-test was applied to compare normally distributed continuous data samples.

P<0.05 determined statistically significant differences. Statistical analyses were performed using the SPSS software V.29.0.0.0 (SPSS). Graphs were constructed in GraphPad Prism V.10.3.1 (GraphPad Software, La Jolla, California, USA).

## Results

### Regional cohort with confirmed SLE

A total of 351 patients from the KLURING cohort were included. The median age at diagnosis of SLE was 38.0 years (IQR 26.0–53.0) and 302 (86.0%) patients were women. Of the 351 patients, 305 (86.9%) had Caucasian origin. Since the launch of the KLURING cohort in 2008, one hundred and seven (30.5%) patients had dropped out, most commonly due to death (47.7%) or due to moving out of the region (29.0%). Data from all patients’ last available visit resulted in a median age of the patients in KLURING of 58.0 years (IQR 42.0–74.0). The median SLE duration for all patients was 16.0 years (IQR 8.0–25.0) and the median SDI score at the last available follow-up visit was 1.0 (IQR 0.0–3.0). The most frequently fulfilled ACR criteria were the presence of ANA (98.9%; 347/351) and arthritis (76.9%; 270/351), followed by haematological disorder (62.4%; 219/351). A detailed description of the patient population is provided in table 1.

**Table 1:** Background variables and clinical phenotypes of patients included in the regional SLE cohort

	All patients(n=351)	SLE without psoriasis(n=339)	SLE with psoriasis(n=12)	P value
**Variables** *				
Female sex, n (%)	302 (86.0)	296 (87.3)	6 (50.0)	0.003
Age at SLE diagnosis (years), median (IQR)	38.0 (26.0–53.0)	37.0 (26.0–53.0)	42.5 (34.0–61.0)	0.3
Caucasian origin, n (%)	305 (86.9)	294 (86.7)	11 (91.7)	1.0
Number of fulfilled 1982 ACR criteria, mean (SD)	4.8 (1.3)	4.8 (1.3)	5.0 (1.4)	0.7
Disease duration (years), median (IQR)	16.0 (8.0–25.0)	16.0 (8.0–25.0)	20.0 (10.5–23.8)	0.7
Ever smoker, n (%)	163 (46.4)	154 (45.4)	9 (75.0)	0.07
BMI (kg/m^2^), median (IQR)	25.8 (8.0–25.0)	25.8 (8.0–25.0)	25.6 (10.5–23.8)	0.9
Clinical phenotypes* **(fulfilled 1982 ACR criteria), n** **(%)**				
Malar rash	134 (38.2)	130 (38.3)	4 (33.3)	1.0
Discoid lupus	54 (15.4)	51 (15.0)	3 (25.0)	0.4
Photosensitivity	182 (51.9)	173 (51.0)	9 (75.0)	0.1
Oral ulcers	45 (12.8)	45 (13.3)	0 (0.0)	0.4
Arthritis	270 (76.9)	259 (76.4)	11 (91.7)	0.3
Serositis	123 (35.0)	119 (35.1)	4 (33.3)	1.0
Renal disorder	93 (26.5)	89 (26.3)	4 (33.3)	0.5
Neurological disorder	20 (5.7)	19 (5.6)	1 (8.3)	0.5
Haematological disorder	219 (62.4)	212 (62.5)	7 (58.3)	0.8
Immunological disorder	200 (57.0)	195 (57.5)	5 (41.7)	0.4
ANA†	347 (98.9)	335 (98.8)	12 (100.0)	1.0

* Data from last available visit.

† Immunofluorescence microscopy.

ACR, American College of Rheumatology; BMI, body mass index.

### Patients with psoriasis in KLURING

In the KLURING cohort, 15 individuals with ICD-10 code L40 (psoriasis) were identified. Three of those cases were excluded as the diagnosis of psoriasis could not be validated through in-depth review of medical records. This eventually resulted in 12 individuals with confirmed coexisting psoriasis (3.4%; 12/351). For three of the patients (25%), the diagnosis of psoriasis was obtained before the diagnosis of SLE. Furthermore, the mean disease duration of psoriasis with coexisting SLE was 9.1 years (range 2–19, SD 5.2). The mean age at psoriasis diagnosis was 54.7 years (range 36–74, SD 11.5), and six out of 12 patients were men (50.0%). There was a higher proportion of men in the subgroup of patients with SLE with comorbid psoriasis compared with the non-psoriasis SLE subgroup (p=0.003). Nine of the 12 patients were former or active smokers (75.0%). History of tobacco smoking was more common among patients with psoriasis, but this difference did not reach statistical significance (p=0.07).

The most common types of psoriasis were plaque psoriasis (66.7%; 8/12), followed by palmoplantar pustulosis (33.3%; 4/12). All 12 individuals were prescribed topical treatment for psoriasis, four combined with methotrexate (33%). Three patients (25.0%) had been treated with biologics (belimumab and/or rituximab) for SLE. We found no significant differences comparing medical treatment for patients with or without coexisting psoriasis. Positivity for anti-U1-RNP (66.7%; 8/12), anti-SSA (58.3%; 7/12) and anti-SSB (58.3%; 7/12) was frequent among patients with comorbid psoriasis. Table 2 summarises data on serological biomarkers, including comparisons between patients with and without psoriasis.

**Table 2:** Serological characteristics of subjects included in the regional SLE cohort

	All patients (n=351)	SLE without psoriasis (n=339)	SLE with psoriasis (n=12)	P value
**Variables** * **, n (%)**				
Anti-dsDNA (+)	176 (50.1)	173 (51.0)	3 (25.0)	0.09
Anti-Sm (+)	30 (8.5)	29 (8.6)	1 (8.3)	1.0
Anti-U1-RNP (+)	136 (38.7)	128 (37.8)	8 (66.7)	0.07
Anti-SSA (+)	168 (47.9)	161 (47.5)	7 (58.3)	0.6
Anti-SSA/Ro52 (+)	130 (37.0)	124 (36.6)	6 (50.0)	0.4
Anti-SSA/Ro60 (+)	127 (36.2); n=349	120 (35.6); n=337	7 (58.3)	0.1
Anti-SSB (+)	85 (24.2)	82 (24.2)	3 (25.0)	1.0
aCL				
IgG (+)	79 (22.5)	78 (23.0)	1 (8.3)	0.3
IgA (+)	24 (6.8); n=232	23 (10.3); n=224	1 (12.5); n=8	0.4
IgM (+)	23 (6.6); n=349	22 (6.5); n=337	1 (8.3)	0.6
aβ2GPI				
IgG (+)	59 (16.8); n=349	58 (17.2); n=337	1 (8.3)	0.7
IgA (+)	36 (10.3); n=232	34 (15.2); n=224	2 (25.0); n=8	0.4
IgM (+)	43 (12.3); n=349	42 (12.5); n=337	1 (8.3)	1.0
Lupus anticoagulant test (+)	99 (28.2); n=318	95 (30.9); n=307	4 (36.4); n=11	0.7
Antiphospholipid syndrome†	57 (16.2)	55 (16.2)	2 (16.7)	1.0
Anti-CCP (+)	18 (5.1); n=254	18 (7.3); n=246	0 (0.0); n=8	N/A
Rheumatoid factor (+)	36 (10.3); n=153	35 (23.6); n=148	1 (20.0); n=5	1.0
Low C3	64 (18.2)	64 (18.9)	0 (0.0)	N/A
Low C4	157 (44.7)	151 (44.5)	6 (50.0)	0.8

* Data from last available visit (ever positivity).

† According to the Sydney criteria.^53^ In case of missing values, the total number of observations is indicated.

aCL, anticardiolipin antibodies; aβ2GPI, anti-β2-glycoprotein-I; CCP, cyclic citrullinated peptide; dsDNA, double-stranded DNA; IgA, immunoglobulin A; IgG, immunoglobulin G; IgM, immunoglobulin M; N/A, not applicable; Sm, Smith antigen; SSA, Sjögren’s syndrome A; SSB, Sjögren’s syndrome B; U1-RNP, U1-ribonucleoprotein.

### Disease activity, organ damage and PROMs

**Figure 1:**
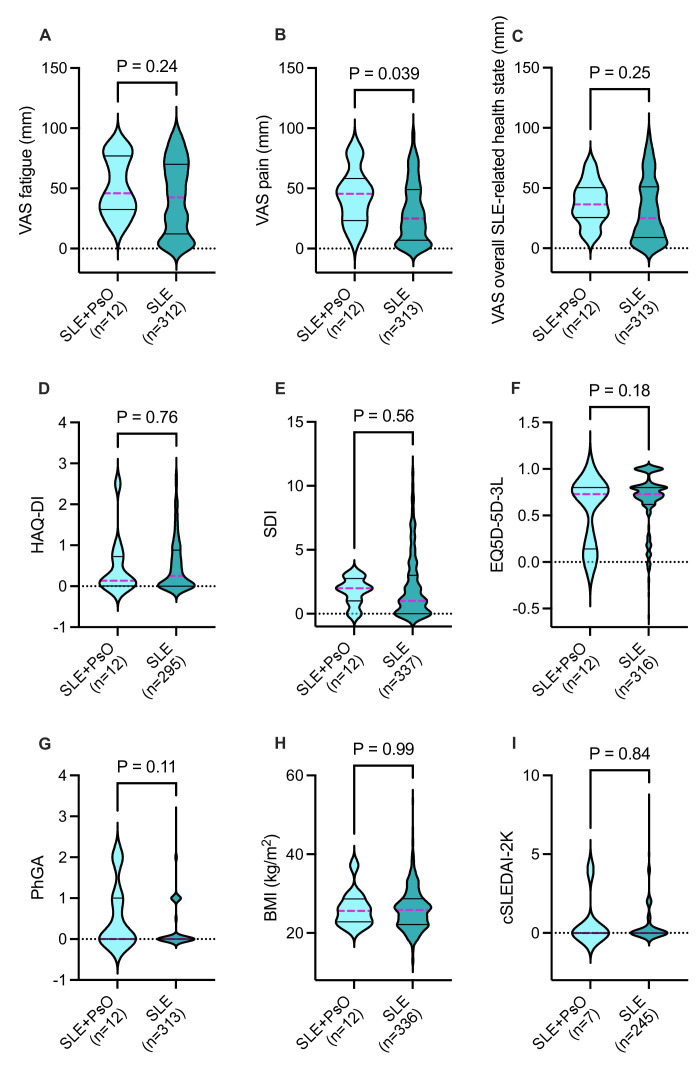
(A–I) Comparisons of patient-reported outcomes and some clinical parameters between patients with SLE with and without coexistent psoriasis (PsO). BMI, body mass index; cSLEDAI-2K, clinical version of SLE Disease Activity Index 2000; EQ-5D-3L, 3-level version of EQ-5D questionnaire; HAQ-DI, Health Assessment Questionnaire Disability Index; PhGA, Physician’s Global Assessment; SDI, Systemic Lupus International Collaborating Clinics/American College of Rheumatology Damage Index; VAS, visual analogue scale.

No significant difference in SLE disease activity, assessed by cSLEDAI-2K, was found between patients with SLE with and without psoriasis over the last three available visits when comparing the median cSLEDAI-2K value (median cSLEDAI-2K, IQR 0.0–1.5 vs 0.0–0.0, respectively, p=0.5). Accumulated organ damage assessed by SDI did not differ significantly between the two groups at the last available visit (p=0.6). For patients with SLE who developed psoriasis (n=9), the mean SDI score was 1.0 (range 0–3, SD 1.1) at the time of psoriasis diagnosis. Two years before the diagnosis, the mean SDI score was 0.9 (n=6, range 0–3, SD 1.2), and 2 years after the diagnosis, the mean score was 1.5 (n=9, range 0–3, SD 1.2). Patients with SLE and comorbid psoriasis reported higher values on the VAS pain scale (median 45.5 mm, IQR 23.3–58.3) compared with patients without psoriasis (median 27.0 mm, IQR 7.0–50.5, p<0.04). Other PROMs yielded no clear differences between the two groups (figure 1).

### Nationwide cohort

A total of 7490 individuals with SLE living in Sweden on 31 December 2022 were identified in the NPR, of which 6497 (86.7%) were women. The mean age as of 31 December 2022 was 59.0 years (range 18–104, SD 17.5). The number of patients with coexisting psoriasis was 367 (4.9%). Of these, 150 (40.9%) received the diagnosis of psoriasis before the diagnosis of SLE. The mean SLE disease duration was 16.5 years (range 1–54, SD 10.8). For those with psoriasis, the mean disease duration with psoriasis was 13.3 years (range 0–40, SD 7.8). Men were proportionally more common in the group of patients with SLE with comorbid psoriasis (p=0.03). In the sensitivity analysis including at least one dispensation of a topical vitamin D analogue, the number of patients with SLE with comorbid psoriasis was 442 (5.9%). Results were otherwise similar (online supplemental table S1).

Regarding pharmacological therapy, patients with SLE and comorbid psoriasis had been dispensed methotrexate and biologics to a greater extent compared with those without comorbid psoriasis. A more detailed description of patient characteristics including therapies is provided in table 3.

**Table 3:** Characteristics of adult patients living with SLE in Sweden identified from the Swedish National Patient Register

	All patients(n=7490)	SLE without psoriasis(n=7123)	SLE with psoriasis(n=367)	P value
**Variable**				
Female sex, n (%)	6497 (86.7)	6193 (86.9)	304 (82.3)	0.03
SLE diagnosis, year interval				
1969–2000, n (%)	1134 (15.1)	1075 (15.1)	59 (16.1)	
2001–2010, n (%)	3067 (40.9)	2908 (40.8)	159 (43.3)	
2011–2022, n (%)	3289 (43.9)	3140 (44.1)	149 (40.6)	
Age in 2022, mean (SD, range), years	59.0 (17.5, 18–104.2)	58.8 (17.5, 18.0–104.3)	63.6 (15.9, 22.7–95.6)	<0.001
Disease duration SLE, mean (SD, range), years	16.5 (10.8, 1.0–53.8)	16.5 (10.8, 1.0–53.8)	17.1 (10.6, 1.0–52.9)	0.25
Disease duration psoriasis, mean (SD, range), years	N/A	N/A	13.3 (7.8, 0.3–39.8)	
**Immunomodulating therapies, dispensed year 2005–2022**				
Antimalarials, n (%)	5711 (76.2)	5446 (76.5)	265 (70.5)	0.06
Methotrexate, n (%)	1903 (25.4)	1735 (24.4)	168 (44.7)	<0.0001
TNF inhibitors, n (%)	214 (2.9)	180 (2.5)	34 (9.0)	<0.0001
Other biologics*, n (%)	346 (4.6)	311 (4.4)	35 (9.3)	<0.0001
JAK inhibitors, n (%)	51 (0.7)	42 (0.6)	9 (2.4)	0.0007
Other immunosuppressants†, n (%)	3401 (45.4)	3233 (45.4)	168 (44.7)	0.9
**Immunomodulating therapies, dispensed 2021–2022**				
Antimalarials, n (%)	4030 (53.8)	3869 (54.3)	161 (43.9)	<0.0001
Methotrexate, n (%)	836 (11.2)	764 (10.7)	72 (19.6)	<0.0001
TNF inhibitors, n (%)	76 (1.0)	66 (0.9)	10 (2.7)	0.004
Other biologics*, n (%)	249 (3.3)	44 (0.6)	21 (5.7)	<0.0001
JAK inhibitors, n (%)	34 (0.5)	0 (0.0)	7 (1.9)	N/A
Other immunosuppressives†, n (%)	1842 (24.6)	1765 (24.8)	78 (21.3)	0.1

* Subgroup consisting of abatacept, anifrolumab, bimekizumab, belimumab, brodalumab, guselkumab, intravenous immunoglobulin, ixekizumab, risankizumab, rituximab, secukinumab, tildrakizumab and ustekinumab.

† Subgroup consisting of azathioprine, cyclosporin A, cyclophosphamide, leflunomide, everolimus, mycophenolic acid, sirolimus, sulfasalazine, tacrolimus and voclosporin.

JAK, Janus kinase; N/A, not applicable; TNF, tumour necrosis factor.

## Discussion

This study aimed to evaluate and describe the coexistence of psoriasis in a well-characterised SLE cohort in Sweden and further expand the exploration using Swedish NPR data. Additionally, we compared patients with SLE with and without coexisting psoriasis for clinical characteristics and serological markers. The estimate of comorbid psoriasis in the regional SLE cohort was 3.4% and the corresponding nationwide estimate from the NPR was moderately higher (4.9%). However, the fact that psoriasis could not be confirmed in 3/15 patients (who carried the ICD code for psoriasis) in the regional cohort indicates that our finding of 4.9% in the NPR probably is an overestimation. Nevertheless, our findings from Sweden confirm data reported from the Toronto Lupus Cohort.^19^

The prevalence of psoriasis, as well as of SLE, in adults in Canada is comparable to the numbers reported in Sweden.^2333^,^35^ Results from the present study indicate that the prevalence of psoriasis in patients with SLE is at least twice as high as that reported for the general Swedish population.^17^ In the regional cohort, the diagnosis of psoriasis had been obtained before the onset of SLE in 25% of the cases and, in the NPR, in more than 40% of the subjects. Nevertheless, we acknowledge that both overestimation and underestimation may occur because of the phenotypical similarities between psoriasis and variants of subacute cutaneous and discoid lupus erythematosus.

A study from the USA investigated the differences between hospitalised individuals with SLE with and without psoriasis as a comorbidity and found 0.7% having coexisting diagnoses.^36^ There is uncertainty about how our results from Sweden compare to other parts of the world, due to the absence of global data on the coexistence of psoriasis in SLE populations. This highlights the importance of this study and the need for further investigation to better define the epidemiological coexistence of SLE and psoriasis. Psoriasis is known to affect both sexes equally, whereas SLE is recognised to primarily affect women. The regional data in our study showed an equal distribution of sexes among patients with SLE with comorbid psoriasis. Furthermore, in the nationwide cohort with larger numbers, we observed a significant over-representation of male patients with comorbid psoriasis compared with those without psoriasis. Notably, this sex bias was also reported in the Canadian study.^19^

According to our findings, no difference in organ damage, measured by the SDI, accrued by the last available follow-up visit, was found between patients with SLE with and without comorbid psoriasis. The study from the USA of hospitalised patients with SLE with and without psoriasis included a total of 20 630 hospitalisations with SLE as the principal diagnosis. The authors of the study found no difference in hospital outcomes (eg, length of stay, total hospital cost or acute kidney injury) between patients with coexisting psoriasis and those with non-psoriasis SLE.^36^ Accordingly, we found no significant differences between the two groups related to clinical phenotypes or immunological characteristics.

A majority of the people suffering from chronic diseases experience difficulties in daily life activities due to their conditions. Patients with psoriasis constitute no exception; a systematic review found an association between psoriasis and poor patient-reported health-related quality of life.^37^ In the present study, patients with SLE with coexistent psoriasis reported more pain on the VAS pain scale compared with those without psoriasis. Therefore, it may be especially important to provide patient education and other pharmacological and non-pharmacological support to this subgroup of patients with SLE to help reduce pain and hopefully improve their quality of life.^38 39^ One could speculate that multimorbidity in general may contribute to poorer reports on PROMs.^40^ To this end, it is worth noting that no differences were observed in SDI scores between the two subgroups of patients with SLE in the regional cohort of the present study.

Managing SLE and psoriasis together requires careful considerations. Ultraviolet radiation, a common non-pharmacological strategy for mild psoriasis, is contraindicated in SLE since it can induce interferon signalling, rash and hypersensitivity reactions.^41^ Of note, 75% of the patients with SLE with comorbid psoriasis in our regional SLE cohort fulfilled the ACR criterion for photosensitivity. Most patients with SLE have been exposed to glucocorticoids long term, and considering their potential adverse effects,^42^ the maintenance daily dose should not exceed 5 mg of a prednisone equivalent.^14^ In psoriasis, systemic treatment with glucocorticoids is traditionally discouraged due to the risk of psoriatic flares, especially in patients with palmoplantar pustulosis. Nevertheless, glucocorticoids are still frequently prescribed, and flares are reported to be infrequent.^43^

Methotrexate is commonly used to manage skin and joint involvement in SLE, and it is also frequently used in psoriasis. Following hydroxychloroquine, methotrexate was the second most common drug used in patients with comorbid psoriasis in the nationwide cohort. However, hydroxychloroquine use entails a potential risk of psoriatic flares.^16^ Biological therapies for psoriasis have high efficacy and comprise three main targeting categories: TNF inhibitors, interleukin-12 (IL-12) and IL-23 inhibitors, and IL-17 inhibitors.^44^ For SLE, two biological agents are approved to date: belimumab, targeting the soluble counterpart of B cell activating factor belonging to the TNF family, and anifrolumab, targeting the type I interferon receptor.^45^ In KLURING, 25% of the patients with SLE and comorbid psoriasis had been or were currently on biologics for managing their SLE, not their psoriasis. In the nationwide cohort, biologics were more common among patients with comorbid psoriasis. The intracellular Janus kinase (JAK)-STAT cascade plays a role both in SLE and psoriasis, and several JAK inhibitors have been trialled in both diseases.^46^,^48^ Recently, a phase II randomised controlled trial (RCT) reported data favouring the use of upadacitinib in SLE,^49^ a drug approved for treating PsA.^50^ The tyrosine kinase 2 (TYK2) inhibitor deucravacitinib, approved for treating psoriasis,^50^ is currently being trialled in a phase III RCT for SLE after meeting its primary and key secondary endpoints in a phase II trial.^51^ Ustekinumab and IL-17 inhibitors are commonly used in psoriasis and have also been trialled for SLE.^44 45^ While the future for JAK and TYK2 inhibitors in SLE is currently unknown, this therapeutic strategy is of high interest,^52^ particularly for patients with comorbid psoriasis.

Our study has limitations. The digital data extraction from the regional cohort includes only SLE cases from 2008 onwards, which is due to the introduction of the digitalisation of medical charts in the County of Östergötland. There are also limitations pertaining to the use of data from the NPR. The diagnoses of SLE and psoriasis in patients with relevant ICD codes for the two diseases are not individually confirmed with classification criteria through chart review. In the NPR, we are likely to underestimate the prevalence of psoriasis since data from primary care visits (where a large proportion of patients with psoriasis are diagnosed and cared for in Sweden) are missing. The psoriasis diagnoses identified in the nationwide data likely represent more severe disease in need of specialised care. To address potential misclassification of psoriasis, in a sensitivity analysis we broadened the definition of psoriasis to include anyone who had a dispensation for a topical vitamin D analogue, which increased the prevalence of comorbid psoriasis to 5.9%. Also, the PDR includes pharmacy dispensing data and not drugs administered in the hospital (eg, intravenous drugs). The relatively small sample size of patients with SLE and coexisting psoriasis in the KLURING cohort limited our power in statistical calculations, but the nationwide data offered better power and are probably more generalisable. One of the major strengths of our study was that all patients from the regional cohort were well characterised and their diagnoses of SLE and psoriasis were definite. In KLURING, all SLE cases with an ICD code for psoriasis were individually reviewed using the patients’ medical charts to validate the diagnosis of psoriasis.

In summary, the prevalence of psoriasis in patients with SLE residing in Sweden is at least 4%. Subjects with SLE and comorbid psoriasis were proportionally more often men, reported more pain on a VAS pain scale and were more frequently prescribed methotrexate and biologics compared with patients without psoriasis.

## Supplementary material

10.1136/lupus-2025-001504 online supplemental file 1

## Data Availability

Data are available upon reasonable request.
