# Supplementary material for: Comorbid psoriasis in systemic lupus erythematosus: a cohort study from a tertiary referral centre and the National Patient Register in Sweden
Source: Lupus Sci Med. 2025 Jun 4;12(1):e001504. doi: 10.1136/lupus-2025-001504 (PMC12142130; doi:10.1136/lupus-2025-001504)
Supplement: online supplemental file 1 [file lupus-12-1-s001.docx]

**Supplementary Table S1.** Characteristics of adult patients living with SLE in Sweden identified from the Swedish National Patient Register, psoriasis defined as at least 1 visit listing an ICD code for psoriasis or at least one dispensation of a topical vitamin D analogue (ATC code D05AX02 or D05AX52).

|  | | **All patients** (*n*=7490) | **SLE without psoriasis**  (*n*=7048) | **SLE with psoriasis**  (*n*=442) |
| --- | --- | --- | --- | --- |
| **Variable** | | | | |
| Female sex, *n* (%) | | 6497 (86.7) | 6132 (87.0) | 365 (82.6) |
| SLE diagnosis, year interval | | | | |
|  | 1969–2000, *n* (%) | 1134 (15.1) | 1062 (15.1) | 72 (16.3) |
|  | 2001–2010, *n* (%) | 3067 (40.9) | 2879 (40.8) | 189 (42.8) |
|  | 2011–2022, *n* (%) | 3289 (43.9) | 3108 (44.1) | 181 (41.0) |
| Age in 2022, mean (SD, range), years | | 59.0 (17.5, 18–104.2) | 58.7 (17.5, 18.0–104.3) | 63.8 (16.2, 20.7–95.9) |
| Disease duration SLE, mean (SD, range), years | | 16.5 (10.8, 1.0–53.8) | 16.5 (10.8, 1.0–53.8) | 17.0 (10.6, 1.0–52.9) |
| Disease duration psoriasis, mean (SD, range), years | | N/A | N/A | 12.6 (7.6, 0.3–39.8) |
| Psoriasis diagnosis before SLE diagnosis, *n* (%) | | N/A | N/A | 176 (39.8) |
| Both psoriasis ICD code and vitamin D analogue, *n* (%) | | N/A | N/A | 145 (32.8) |
| Only psoriasis ICD code, *n* (%) | | N/A | N/A | 222 (50.2) |
| Only vitamin D analogue, *n* (%) | | N/A | N/A | 75 (17.0) |
| Any vitamin D analogue, *n* (%) | | N/A | N/A | 202 (49.8) |
| **Immunomodulating therapies, dispensed year 2005–2022** | | | | |
| Antimalarials, *n* (%) | | 5711 (76.2) | 5391 (76.5) | 320 (72.4) |
| Methotrexate, *n* (%) | | 1903 (25.4) | 1713 (24.3) | 190 (43.0) |
| TNF inhibitors, *n* (%) | | 214 (2.9) | 179 (2.5) | 35 (7.9) |
| Other biologics^1^, *n* (%) | | 346 (4.6) | 309 (4.4) | 37 (8.4) |
| JAK inhibitors, *n* (%) | | 51 (0.7) | 42 (0.6) | 9 (2.0) |
| Other immunosuppressants^2^, *n* (%) | | 3401 (45.4) | 3202 (45.4) | 199 (45.0) |
| **Immunomodulating therapies, dispensed 2021–2022** | | | | |
| Antimalarials, *n* (%) | | 4030 (53.8) | 3839 (54.5) | 191 (43.2) |
| Methotrexate, *n* (%) | | 836 (11.2) | 751 (10.7) | 85 (19.2) |
| TNF inhibitors, *n* (%) | | 76 (1.0) | 66 (0.9) | 10 (2.3) |
| Other biologics^1^, *n* (%) | | 249 (3.3) | 226 (3.2) | 23 (5.2) |
| JAK inhibitors, *n* (%) | | 34 (0.5) | 27 (0.4) | 7 (1.6) |
| Other immunosuppressives^2^, *n* (%) | | 1842 (24.6) | 1768 (25.1) | 94 (21.3) |

JAK, janus kinase; N/A, not applicable; SD, standard deviation; SLE, systemic lupus erythematosus; TNF, tumor necrosis factor.

^1^Subgroup consisting of abatacept, anifrolumab, bimekizumab, belimumab, brodalumab, guselkumab, intravenous immunoglobulins, ixekizumab, risankizumab, rituximab, secukinumab, tildrakizumab, ustekinumab. ^2^Subgroup consisting of azathioprine, cyclosporin A, cyclophosphamide, leflunomide, everolimus, mycophenolic acid, sirolimus, sulfasalazine, tacrolimus and voclosporin.
